# A Rising Star of the Multimarker Panel: Growth Differentiation Factor-15 Levels Are an Independent Predictor of Mortality in Acute Heart Failure Patients Admitted to an Emergency Clinical Hospital from Eastern Europe

**DOI:** 10.3390/life12121948

**Published:** 2022-11-22

**Authors:** Radu-Stefan Miftode, Daniela Constantinescu, Corina-Maria Cianga, Antoniu-Octavian Petris, Irina-Iuliana Costache, Ovidiu Mitu, Ionela-Larisa Miftode, Ivona Mitu, Amalia-Stefana Timpau, Stefania-Teodora Duca, Alexandru-Dan Costache, Petru Cianga, Ionela-Lacramioara Serban

**Affiliations:** 1 Department of Internal Medicine I (Cardiology), Faculty of Medicine, University of Medicine and Pharmacy “Gr. T. Popa”, 700115 Iasi, Romania; 2 Department of Immunology, Faculty of Medicine, University of Medicine and Pharmacy “Gr. T. Popa”, 700115 Iasi, Romania; 3 Department of Infectious Diseases, Faculty of Medicine, University of Medicine and Pharmacy “Gr. T. Popa”, 700115 Iasi, Romania; 4 Department of Morpho-Functional Sciences II, University of Medicine and Pharmacy “Gr. T. Popa”, 700115 Iasi, Romania; 5 Department of Cardiovascular Rehabilitation, Faculty of Medicine, University of Medicine and Pharmacy “Gr. T. Popa”, 700115 Iasi, Romania

**Keywords:** GDF-15, acute heart failure, multimarker, mortality predictor

## Abstract

(1) Background: Acute heart failure (HF) represents one of the most common yet extremely severe presentations in emergency services worldwide, requiring prompt diagnosis, followed by an adequate therapeutic approach, and a thorough risk stratification. Natriuretic peptides (NPs) are currently the most widely implemented biomarkers in acute HF, but due to their lack of specificity, they are mainly used as ruling-out criteria. Growth differentiation factor-15 (GDF-15) is a novel molecule expressing different pathophysiological pathways in HF, such as fibrosis, remodeling, and oxidative stress. It is also considered a very promising predictor of mortality and poor outcome. In this study, we aimed to investigate the GDF-15’s expression and particularities in patients with acute HF, focusing mainly on its role as a prognosis biomarker, either per se or as part of a multimarker panel. (2) Methods: This unicentric prospective study included a total of 173 subjects, divided into 2 subgroups: 120 patients presented in emergency with acute HF, while 53 were ambulatory-evaluated controls with chronic HF. At admission, all patients were evaluated according to standard clinical echocardiography and laboratory panel, including the assessment of GDF-15. (3) Results: The levels of GDF-15 were significantly higher in patients with acute HF, compared to controls [596 (305–904) vs. 216 (139–305) ng/L, *p* < 0.01]. GDF-15 also exhibited an adequate diagnostic performance in acute HF, expressed as an area under the curve (AUC) of 0.883 [confidence interval (CI) 95%: 0.828–0.938], similar to that of NT-proBNP (AUC: 0.976, CI 95%: 0.952–1.000), or troponin (AUC: 0.839, CI 95%: 0.733–0.944). High concentrations of GDF-15 were significantly correlated with mortality risk. In a multivariate regression model, GDF-15 was the most important predictor of a poor outcome, superior to NT-proBNP or troponin. (4) Conclusions: GDF-15 proved to be a reliable tool in the multimarker assessment of patients with acute HF. Compared to the gold standard NT-proBNP, GDF-15 presented a similar diagnostic performance, doubled by a significantly superior prognostic value, making it worth being included in a standardized multimarker panel.

## 1. Introduction

Heart failure (HF) is a polymorphic, multifactorial clinical syndrome that represents the final pathway for a plethora of cardiovascular pathologies. Its various etiologies and risk factors, doubled by the rise in life expectancy and the growth of the aging population worldwide, have outlined HF’s central role in the so-called “cardiac pandemic,” a phenomenon associated with considerable morbidity, mortality, and a major burden on health systems [1,2].

Acute HF represents a common yet complex presentation in emergency departments, requiring a high index of suspicion in order to differentiate it from other causes of sudden-onset dyspnea, and also a prompt diagnosis of the underlying cardiovascular substrate, followed by adequate therapeutic management. Not only is the initial diagnosis essential, but also the risk stratification in the first hours after hospitalization, acute HF being characterized by high mortality rates (both in-hospital and after discharge) and frequent readmissions, with a generally poor prognosis [3,4,5].

Under these circumstances, diagnosing acute HF as soon as possible after the first medical contact is of paramount importance not only for the short-term prognosis but also for the long-term survival, quality of life, socioeconomic reintegration, and psycho-affective profile of the patients presenting this pathology [6,7]. In this regard, cardiac biomarkers are increasingly used on a large scale in acute HF, especially in the emergency room, as they prove to be fast, relatively reliable, non-invasive, reasonably priced, and easy to interpret diagnostic tools [8].

Natriuretic peptides (NPs) are currently the most used biomarkers. The 2021 HF guidelines of the European Society of Cardiology (ESC) recommending the assessment of these molecules to rule out acute HF, at specific thresholds: <100 pg/mL for B-type natriuretic peptide (BNP) and 300 pg/mL for its N-terminal prohormone (NT-proBNP). Despite their high sensitivities, NPs have a rather low specificity in confirming the diagnosis of acute HF, their levels being influenced by age, and multiple other comorbidities, such as myocardial ischemia, inflammation, sepsis, renal dysfunction, obesity, pulmonary embolism, arrhythmias, or stroke [2]. As for risk stratification, the same ESC guidelines do not recommend measuring NPs for prognosis evaluation in patients with acute HF.

All these aspects render NPs an imperfect gold standard and draw attention to the importance of researching new molecules, at least as sensitive, but more specific, cost-effective, and, in addition, with a solid prognostic role in predicting negative outcomes. Growth differentiation factor-15 (GDF-15)—a ubiquitous cytokine—could be a promising option, as it is poorly expressed in healthy individuals, but it markedly increases during stressful conditions, such as left ventricular injury, volume, and pressure overload, or remodeling. More and more literature data suggest its possible implementation as a useful biomarker in HF [9,10]. In this regard, the results of various experimental studies are cited, indicating that GDF-15 confers protection against myocardial injury due to its antihypertrophic, anti-inflammatory, and antiapoptotic properties, being overexpressed in certain pathological conditions [10]. Furthermore, several authors have emphasized its prognostic role, given the significant correlations between elevated serum levels of GDF-15 and increased mortality, possibly being a stronger predictor than NT-proBNP [11,12]. An interesting section of the Framingham study assessed 85 cardiac biomarkers (including GDF-15) in approximately 3500 patients, noting that in a multivariate analysis, GDF-15 was the only biomarker that was significantly associated with the three important end-points: atherosclerotic pathologies, occurrence of HF, and mortality rate [13]. In another recent extensive study performed in patients without pre-existing cardiovascular (CV) pathologies, GDF-15 was an independent predictor of CV and all-cause mortality (RR 1.5; 95% CI 1.3–1.8), values superior to those of BNP (RR 1.3; 95% CI 1.2–1.5) [14].

Structurally, GDF-15 is initially synthesized as a prohormone, which subsequently undergoes dimerization, resulting in the pro-GDF-15 dimer. The latter is further cleaved at a specific site, forming a C-terminal dimeric protein consisting of 112 amino acids responsible for the biological effects of GDF-15- and a pro-peptide. Furthermore, the dimeric protein is secreted into the extracellular matrix and can be determined in serum by standard immunological assays, representing, in fact, the actual biomarker. Intracellularly, GDF-15 exhibits a structural polymorphism based on the coexistence of several isoforms with different molecular weights, ranging from 30 kDa to 80 kDa, that act as regulators of transcriptional mechanisms [15,16].

Unlike natriuretic peptides, which are secreted by cardiac myocytes in response to wall stress (due to pressure or volume overload), several studies have demonstrated that GDF-15 also represents the expression of several other pathophysiological pathways highly incriminated in HF, such as inflammation, oxidative stress, fibrosis, and even reparative bioprocesses [10,17,18].

**Figure 1:**
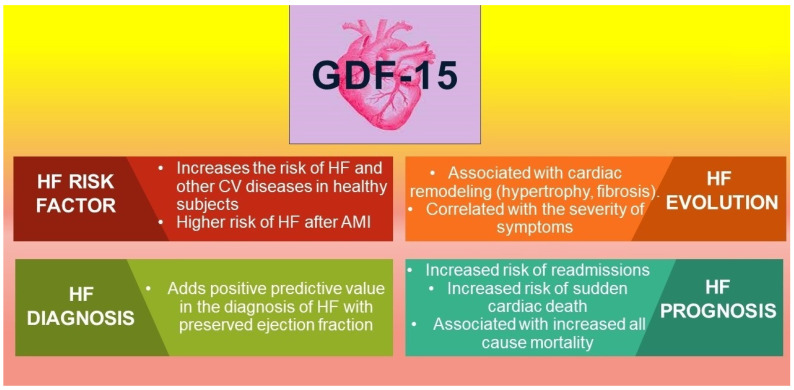
GDF-15 and its implications as a biomarker in HF. Legend: HF-heart failure; CV-cardiovascular; AMI-acute myocardial infarction.

The implications of GDF-15 as a biomarker for HF are depicted in Figure 1.

In this study, we aimed to investigate the intrinsic diagnostic and prognostic capacity of GDF-15 in patients presenting in emergency units with acute HF, as previous studies focused mainly on chronic HF. Moreover, another goal of this research was to evaluate the correlations between GDF-15 and the so-called classic biomarkers (NT-proBNP, high-sensitive troponin) and the opportunity to integrate them in a multimarker model with enhanced prognostic value.

## 2. Materials and Methods

### 2.1. Study Design, Patients, and Investigations

We conducted a prospective study that included 120 consecutively enrolled patients admitted for acute HF in St. Spiridon Emergency County Hospital (Iasi) between January 2021 and June 2021, as it is the largest and most relevant medical facility in northeast Romania, a region with more than 4 million inhabitants. We included patients presenting in emergency with sudden-onset or rapidly progressive dyspnea who were admitted to the Cardiology Clinic with a confirmed diagnosis of acute HF, in any of its clinical presentations, as defined by ESC (acute decompensated HF, cardiogenic shock, acute pulmonary edema, isolated right ventricular failure). The clinical diagnosis was based on Framingham criteria, a definite HF diagnosis requiring at least two major, or one major + two minor criteria. To further confirm the diagnosis, all patients underwent a complete echocardiography examination using a GE VividTM V7 ultrasound system (General Electric, Boston, CA, USA).

We excluded the patients who refused to sign the informed consent at admission, the patients with severe or terminal pathologies (e.g., late-stage cancer, active auto-immune diseases, sepsis, or severe concomitant bacterial or viral infections, recent major surgery, on dialysis or on the transplant list), with an NT-proBNP at admission below the ESC-recommended cut-off of 300 pg/mL, or with conditions that rendered impossible an adequate examination (e.g., extreme obesity, chest malformations or poor echocardiographic window). Patients with diagnosed neuropsychiatric disorders, as well as subjects from other vulnerable categories (minors, inmates, refugees, homeless people), were also excluded from the study.

The controls were represented by stable ambulatory patients, who were periodically evaluated, and who presented a previous diagnosis of chronic, compensated HF, without HF-related hospitalizations during the last year.

We performed a detailed anamnesis and a standard physical exam, followed by a thorough assessment of the patients’ medical files, extracting relevant demographical, clinical, and laboratory parameters. For scientific soundness and reproducibility, in our statistical analysis, we used clinical parameters recorded strictly at admission, such as blood pressure, heart rate, or body mass index. A complete standard laboratory test panel (including the classic biomarkers, such as NT-proBNP and hs-troponin) was performed for all the patients during the first hours after admission to the Cardiology Clinic. The cut-off values that were considered within the pathological spectrum were as follows: >300 pg/mL for NT-proBNP, and >29 ng/L for hs-troponin. These concentrations were in accordance with current guidelines [2] and our laboratory assay reference value, respectively.

Comorbidities were either previously documented in the patients’ personal files or diagnosed during hospitalization, complying with the validated diagnostic criteria. For example, just to recall the pathologies with significant cardiovascular impact: obesity was defined as a body mass index (BMI) > 30 kg/m^2^, while the latest ESC Guidelines on the management of arterial hypertension have clearly defined the diagnostic threshold as a blood pressure ≥ 140/90 mmHg [19], Coronary artery disease was diagnosed in patients with a positive history of angina or prior documentation of ischemia suggested by exercise test, echocardiography stress test, or coronary angiography. According to the World Health Organization, anemia was defined as a hemoglobin value < 12 g/dL in women or <13 g/dL in men [20], while diagnosing diabetes mellitus required a fasting blood glucose >126 mg/dL, glycosylated hemoglobin > 6.5%, or current use of antidiabetic medication [21].

For GDF-15’s assessment, we initially collected a small venous blood sample (2 mL) that was centrifuged at 2000 rpm for 20 min in order to separate the serum. Further, serum samples were stored at −80 °C until the day of analysis. Quantification of GDF-15 was performed using ELISA EIAab E2034h kits (EIAAB Science Inc., Wuhan, China), with a detection range of 15–1000 ng/L. The dilution of the standards, the antibody cocktail, and the washing solution were carried out according to the manufacturer’s instructions. The absorbance was read at 450 nm using an Infinite 200 PRO M Plex Microplate Reader (Tecan, Grödig, Austria) after stopping the reaction. The amount of GDF-15 protein in each sample was determined using Magellan Pro v7.4 software (Tecan, Austria) by interpolating the absorbance values in the microwells corresponding to each sample on the standard curve. The coefficient of variation for the duplicate measurements was 5.66% (95% CI 2.192–9.128).

### 2.2. Statistical Analysis

We initially performed the Kolmogorov-Smirnov test for the initial assessment of the variables’ normal distribution within the study population. Subsequently, normally distributed variables were expressed as means ± standard deviation (STD), while non-normally distributed data were presented as medians with interquartile ranges (IQRs: 25–75%). Descriptive data for categorical variables were expressed as frequencies (numbers) and percentages. The differences between variables within the cohort’s various subgroups were assessed either with independent t-test or with Whitney-U test, as appropriate, depending on whether the variables were normally distributed or not. We assessed the correlations between the different parameters by evaluating Pearson’s (for continuous variables) or Spearman’s (for categorical variables) coefficients (r).

The diagnostic performance of the included biomarkers was evaluated by comparing the areas under the curve (AUC) resulting from receiver operating characteristic (ROC) analysis. Relevant diagnostic or prognostic GDF-15 cut-off concentrations were also drawn from the ROC curves. We used the log-rank test to compare the mortality risk within the two subgroups (with serum levels above and below the GDF-15’s high-risk cut-off value), while the Kaplan–Meier curves estimated the subsequent survival distribution.

We performed a linear regression to investigate the interdependence between two variables (biomarkers) that were correlated with each other. Additionally, we also conducted a multivariate regression in order to define a specific multimarker model that comprises at least two biomarkers (independent variables) as predictors that are significantly associated with overall mortality (dependent variable) in patients with acute HF.

We used IBM SPSS v.26 (IBM, Armonk, NY, USA) software to perform the statistical analysis of the collected data. In all cases, a two-tailed *p*-value of 0.05 was considered the threshold for statistical significance.

### 2.3. Ethics

This prospective study was conducted in accordance with the ethical principles stated in the Declaration of Helsinki (revised in 2013). Standard informed consent regarding study purposes and the intended research goals was signed upon admission by every patient, being an integrative part of the personal medical files. All data was anonymously processed, the entire research being approved by the authorized Ethics Committees of both the University of Medicine and Pharmacy “Grigore T. Popa” Iasi (no. 9537/2020), and of the St. Spiridon Emergency Clinical Hospital (no. 41/2020).

## 3. Results

### 3.1. Baseline Characteristics

Demographic characteristics, such as gender and age, were similar between the study group and the controls. The mean length of stay for admitted patients was 10.9 ± 6.8 days, with a median of 9 days (range: 1–41 days, IQR: 8–12.8 days). We recorded 21 fatalities (17.5%), all of which occurred in patients with acute HF. In the same group, we also found a substantially increased prevalence of certain conditions that can contribute to the development of HF—or that can at least trigger an episode of cardiac decompensation—such as alcohol abuse, ischemic heart disease, obesity, or anemia. However, we did not notice significant differences within subgroups concerning other major cardiovascular risk factors, such as smoking, arterial hypertension, and diabetes mellitus. COVID-19 infection is a recent and growingly important predictor for HF, affecting both admitted patients and those evaluated in ambulatory care in roughly similar proportions in this study.

All detailed data are summarized in Table 1.

As expected, a dilated left ventricle (LV) with a more severe systolic dysfunction (expressed as reduced LV ejection fraction) was significantly more prevalent among patients with acute HF (*p* < 0.05). Concerning laboratory profile, we highlighted that hospitalized patients presented a considerably higher serum creatinine, total bilirubin, and potassium, doubled by a lower sodium or HDL-cholesterol, compared to controls. Regarding the therapeutic management of hospitalized patients, compared to their ambulatory counterparts, the use of loop-diuretics and mineralocorticoid receptor antagonists was significantly more prevalent among patients with acute HF (*p* < 0.05), while beta-blockers and inhibitors of the renin–angiotensin–aldosterone system were more commonly administered in control patients, as part of the standard, guideline-recommended therapy in stable heart failure. (Table 2).

### 3.2. Multimarker Panel

We observed that serum concentrations of GDF-15, NT-proBNP, and hs-Troponin were significantly higher in patients with AHF compared to the control group (Table 3). Consequently, we aimed to identify specific correlations between GDF-15 and certain relevant parameters commonly assessed in patients with acute HF (Table 4). Thus, we noted that the concentrations of this novel biomarker did not significantly vary according to LV ejection fraction or to left chamber dimensions but were directly correlated with the increase of LV filling pressures (E/e’) or pulmonary artery systolic pressure.

An important aspect when it comes to the biomarker panel is represented by the strong and positive correlation between GDF-15 and NT-proBNP (R = 0.264, *p* = 0.004), and C-reactive protein (R = 0.177, *p* = 0.047), but not with troponin (R = 0.035, *p* = 0.752). Both systolic and diastolic arterial pressures were negatively correlated with high levels of GDF-15, while some specific behaviors (alcohol abuse, smoking), constitutional aspects (age, gender, BMI), or relevant comorbidities (DM, ischemic heart disease, arterial hypertension) did not significantly influence GDF-15’s serum concentrations. Additionally, GDF-15 exhibited direct and strong correlations not only with clinical aspects suggestive of acute HF (e.g., pulmonary crackles, pulmonary edema) but also with certain predictors of a poor outcome, such as the need for inotropic support, lactate level, or length of hospitalization. Of course, the predictive value of GDF-15 was further ascertained by its significant direct correlations with mortality rates, both in hospital and at 30 days.

### 3.3. Diagnostic Performance of GDF-15

**Figure 2:**
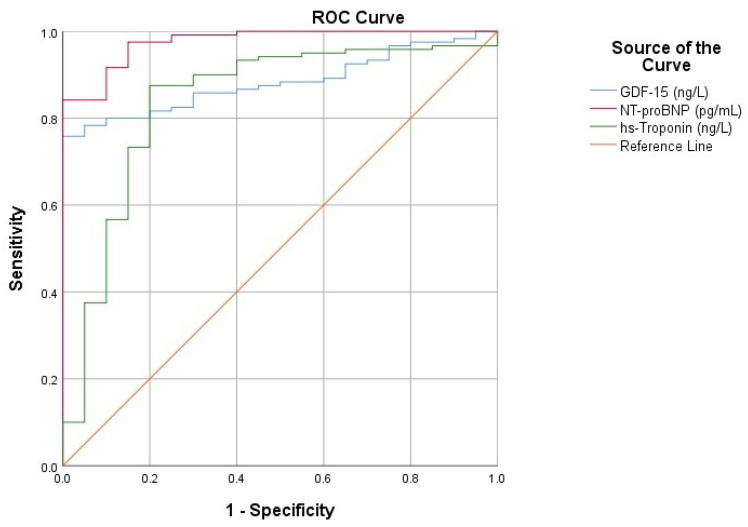
Receiver operating characteristic (ROC) curves for analyzed biomarkers.

Further, to assess the diagnostic performance of GDF-15, we performed a ROC analysis, which exhibited an AUC > 0.8 for all the included biomarkers (Figure 2). This indicated significant potential in positively predicting acute HF in patients presenting with suggestive symptoms. GDF-15’s AUC of 0.883 is similar to that of the extensively used NT-proBNP (AUC = 0.976), which is the current gold standard biomarker in HF. Equally important, the AUC of GDF-15 is also comparable with that of hs-Troponin, suggesting their possible interchangeable use in acute HF, especially in ischemic etiology (Table 5).

**Figure 3:**
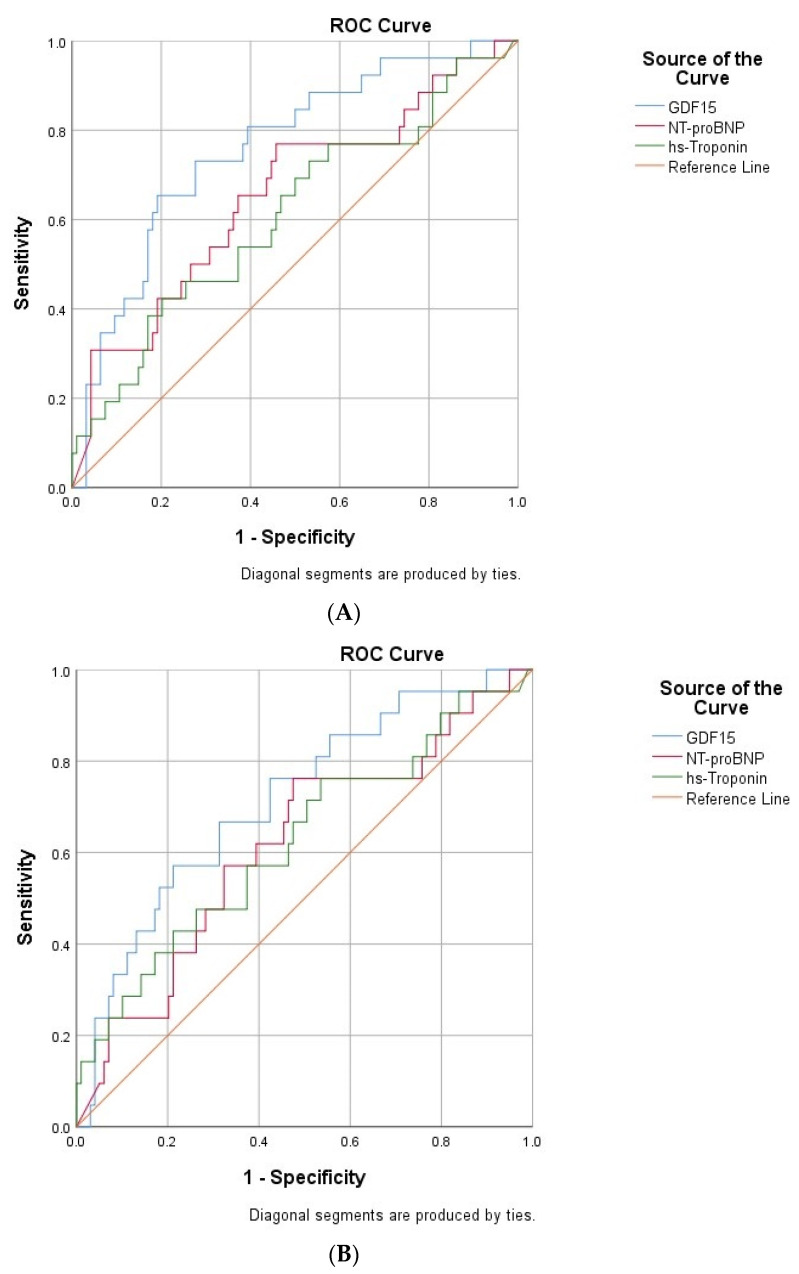
(**A**) ROC curve for the relationship between cardiac biomarkers and 30-day mortality rate. (**B**) ROC curve for the relationship between cardiac biomarkers and in-hospital mortality rates.

Given the previously mentioned positive correlations of GDF-15 with mortality, we drawn additional ROC curves and found thatboth AUC were above 0.7 (0.715 for in-hospital mortality, and 0.760 for 30-days mortality)thus suggesting a significant capacity of GDF-15 in predicting short-term fatalities (*p* < 0.05) (Figure 3A,B). Concerning the classical biomarkers, NT-proBNP and hs-troponin did not show significant predictive abilities either for in-hospital (AUC= 0.617, and 0.626, respectively) or for 30-day mortality rates (AUC = 0.655, and 0.610, respectively).

Following the plotting of the ROC curves, we aimed to extract appropriate cut-off values that can be useful in clinical practice not only as a sensitive and specific diagnostic test but also as a significant mortality predictor. By using the point where sensitivity (Se) and specificity (Sp) are roughly equal, we found that a GDF-15 concentration of 306 ng/L is 75% sensitive and 75% specific in diagnosing acute HF. If we chose Youden’s index (maximum potential effectiveness of a biomarker, represented by the sum of Se and Sp), we found that a slightly higher cut-off (314 ng/L) is equally sensitive (75%), but more specific (77.6%) in predicting acute HF (Table 6). Concerning GDF-15’s prognosis value, we noticed that a cut-off value of 618 ng/L is significantly associated with a poor outcome [OR: 6.47 (CI 95% 2.24–18.66), *p* < 0.001].

**Figure 4:**
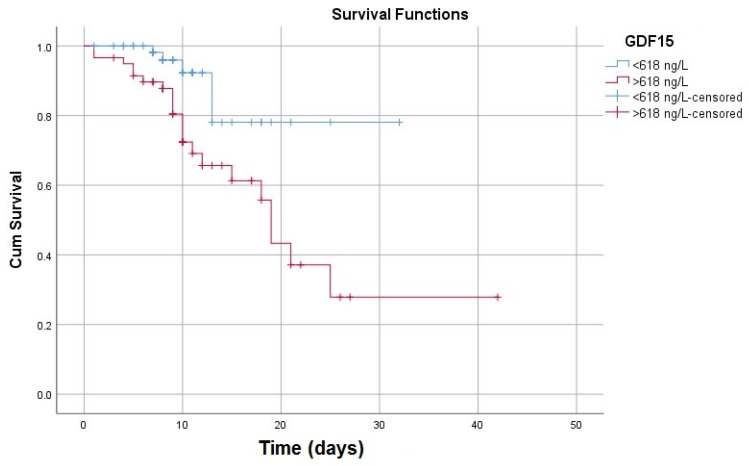
Kaplan–Meier survival curves according to the high-risk cut-off.

Consequently, by plotting the Kaplan-Meier curves according to this concentration, we observed diminished survivability in patients with GDF-15 values higher than 618 ng/L (Figure 4). The further comparison of the mortality risk performed by the log-rank test confirmed a statistically significant difference between the two subgroups, according to the specified 618 ng/L cut-off value (Table 7).

### 3.4. Multimarker Approach

**Figure 5:**
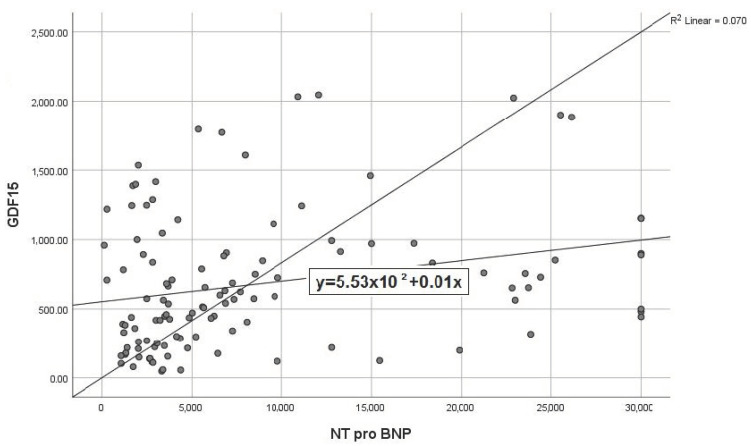
Scatterplot expressing the correlation between GDF-15 and NT-proBNP.

In our study, we observed a linear correlation between the concentrations of GDF-15 and those of NT-proBNP, the latter already being an established marker of severity. The regression equation was y = 553 + 0.01x (R^2^ = 0.070), basically highlighting that every increase of serum NT-proBNP with 1 unit determines a parallel increase of GDF-15 by 0.01% (Figure 5).

Under these circumstances, to evaluate the utility of routine multimarker testing in daily practice, we aimed to highlight the additive value of GDF-15 and NT-proBNP in the prognosis assessment of patients with acute HF. For this purpose, we compared the total mortality rate occurring in patients with acute HF according to a reference point represented by the median serum levels of GDF-15 and NT-proBNP. We observed that the symbiosis between these two biomarkers in predicting fatal events is reflected by a high mortality rate directly dependent on the increase of both molecules but more strongly related to the specific increase of GDF-15 if we take into account the median value of NT-proBNP as a reference. The additive prognostic value of GDF-15 in predicting mortality is substantial in both scenarios, where GDF-15 exceeds the median value, irrespective of the NT-proBNP’s “low” (25% vs. 7.3%) or “high” concentrations (40% vs. 10.5%) (Table 8).

Finally, by the same principle of the multimarker approach, we performed a multivariable regression by which we aimed to identify the association of biomarkers that can best predict mortality in patients with acute HF. Despite that GDF-15 was per se a significant mortality predictor (R = 0.375, R^2^ = 0.141), superior to the dual-marker model composed of NT-proBNP and hs-troponin (R = 0.310, R^2^ = 0.096), we found that a composite test of all three biomarkers can substantially improve the prediction value for a poor outcome in patients with acute HF (R = 0.451, R^2^ = 0.203, *p* < 0.001). Basically, more than 20% of the total mortality rate can be predicted by the variation of GDF-15, hs-troponin, and NT-proBNP. The 1.82 value of the Durbin-Watson test is an appropriate one, within the 1.5–2.5 reference range, which practically excludes the risk of autocorrelation). Although the inclusion of all three biomarkers in the multivariable regression increased the predictive value of the multimarker model, the difference was not statistically significant compared to that of the model that included only GDF-15 and troponin (R= 0.451, R^2^ = 0.192 vs. R = 0.438, R^2^= 0.203, *p* > 0.05), this aspect being explained by the fact that NT-proBNP was no longer a significant predictor of mortality when stepwisely included in the composite model (Table 9).

## 4. Discussion

The severity of its clinical presentation, burdened by high mortality rates and an increasing prevalence worldwide, makes acute HF a hotspot for healthcare systems [22,23]. Currently, the interest of both clinicians and researchers is shifting increasingly toward the extensive use of cardiac biomarkers as a feasible tool for a timely diagnosis and adequate risk stratification. Various molecules, encompassing cytokines (e.g., GDF-15, ST2), enzymes (e.g., hs-troponin), and hormones (e.g., BNP, NT-proBNP), create a vast network of biomarkers that express several molecular pathways involved in the pathogenesis of HF [12]. Despite the fact that NPs are currently widely used in clinical practice, their prognostic value in identifying high-risk patients at admission is rather limited. Nevertheless, several literature data highlighted various molecules as possible surrogate biomarkers in [24,25,26], a growing body of evidence turning the spotlight on GDF-15 and emphasizing that it could be used as a valuable predictor of poor outcome in patients with HF [12,27,28,29].

It is worth mentioning that the vast majority of previous studies exclusively included patients with chronic HF. Under these circumstances, we considered that a head-to-head comparison of GDF-15 serum levels in patients with acute HF versus controls with chronic HF would be particularly relevant. Specifically, we found significantly higher concentrations of GDF-15 in patients presenting in emergencies with acute HF, in accordance with the results recently reported by May et al. [14]. There are several aspects that could explain the differences regarding GDF-15 and its relationship to the severity of HF presentation. Many authors consider that this molecule may reflect disease progression, with serum levels increasing exponentially with worsening New York Heart Association (NYHA) functional class and increased LV remodeling. Basically, GDF-15 levels have been shown to increase right from the subclinical stages of HF, an expression of the pathophysiological continuum involving local and systemic inflammation, oxidative stress, or myocardial ischemia [14,27,30].

According to our results, GDF-15 was inversely correlated with systolic function, but its concentrations were not decisively influenced by LVEF (R= −0.117, *p* = 0.125). The practical importance of this aspect lies in the utility of assessing this biomarker both in symptomatic patients presenting HF with preserved LVEF (HFpEF) and in those with reduced LVEF (HFrEF), given their similar clinical presentation and the ever-increasing prevalence of HF phenotypes characterized by normal systolic function but with diastolic dysfunction and/or elevated LV filling pressures.

This theory was further confirmed in our study by the positive and significant correlations of GDF-15 with clinical aspects suggestive of acute HF (e.g., pulmonary crackles and peripheral edema), irrespective of LVEF. However, the literature data are controversial. On one hand, there are studies that confirm the independent variation of GDF-15 compared to LVEF [9]; on the other hand, there are authors who demonstrated a negative correlation for GDF-15, with significantly higher serum levels in those with impaired LVEF [31]. The vault key addressing this conundrum would be represented by myocardial fibrosis, which represents a constant finding in HF. Several studies have reported substantial correlations between circulating GDF-15 and the degree of cardiac fibrosis. It is assumed that GDF-15 is overexpressed in the presence of enhanced activity of transforming growth factor β (TGF- β), the latter playing a key role in myocardial fibrosis, via fibroblast activation, with subsequent deposition of type III collagen fibers in the extracellular matrix (ECM) [32,33,34]. However, further research is required, as it is not precisely known whether GDF-15 is a consequence or the source of these pathophysiological processes, especially since its serum levels are also correlated with those of matrix metalloproteinases (MMP), which are essential molecules in changing the architecture of ECM due to collagen degradation and turnover, with a subsequent impact on LV stiffness, geometry, and the progression of HF [33]. It is well established that myocardial fibrosis represents the cornerstone of LV remodeling and the consequent hemodynamic alterations. Our study indirectly endorsed this pathway, as GDF-15 was significantly correlated with E/e’, which is not only an important echocardiographic hallmark reflecting increased LV filling pressures (presumably related to cardiac fibrosis) but also a marker of poor prognosis in HF, either individually or as part of more complex tissue Doppler-derived indexes [35].

The prospective nature of our study, conducted in an emergency clinical hospital, creates the premises for the inclusion of patients presenting with various comorbidities, in addition to acute HF. As a marker that mirrors several mechanisms involved in the progression of HF but which are also commonly related to other pathologies, it is obvious that the assessment of GDF-15 in patients with acute HF can be influenced by their non-cardiac comorbidities. Thus, in our study, we noted significant positive correlations between GDF-15 and markers of liver or kidney dysfunction but also negative correlations with serum sodium or total proteins, similar to the findings reported by Chan et al. in 2016 [9]. It is reasonable to assume that the association between increased biological markers suggestive of organ dysfunction and the high levels of GDF-15 may actually be based on the severe endothelial dysfunction induced by GDF-15, with a direct impact on coronary, renal, and hepatic microcirculation [30,36]. Consistent literature data support this hypothesis, demonstrating the negative impact of GDF-15 on microvessels by altering their feedback on both vasoconstrictor stimuli and nitric oxide (NO). Likewise, GDF-15 induces endothelial dysfunction not only through its interference with the normal functioning of NO-dependent vascular systems but also by the exaggerated proliferation of endothelial cells [37]. Simultaneously and somewhat paradoxically, GDF-15 also causes accelerated endothelial senescence by activating pro-oxidant pathways mediated by certain reactive oxygen species (ROS), the final result being the significant alteration of the architecture and functioning of the vascular endothelium, with consecutive cardiac, renal, and hepatic injuries expressed as elevated biological markers [30,38,39,40].

It is worth noting that in our group, GDF-15 correlated negatively with lipid fractions, thus highlighting the possible protective role of this biomarker in atherosclerotic pathology. This hypothesis was supported by a recent study that demonstrated that GDF-15 inhibits lipid accumulation in macrophages treated with oxidized LDL [41] but was refuted by other authors who were demonstrating the overexpression of GDF-15 in the macrophages from atherosclerotic lesions, and the direct correlation with a pro-inflammatory status, specific to atherosclerotic processes [42]. In this regard, we found a significant positive correlation between GDF-15 and inflammation (expressed as a high C-reactive protein), but the exact relationship to atherosclerosis in our study is to be further ascertained, given the fact that C-reactive protein is a non-specific marker, being a validated predictor of poor prognosis in both cardiac [43,44] and non-cardiac pathologies [45]. Of course, decreased lipid fractions could also occur in the setting of liver dysfunction associated with severe HF, an aspect that would also explain the linear increases of GDF-15 in acute HF, and its direct correlations with the markers of liver injury.

Equally important, in our entire cohort, the levels of GDF-15 were not substantially influenced by the presence of traditional cardiovascular risk factors, such as smoking, arterial hypertension, or diabetes mellitus. GDF-15 was not modified by some constitutional aspects, such as age, gender, or BMI—an advantage compared to NPs, as these confounders are commonly incriminated in altering NT-proBNP’s levels [46,47]. Even if the study was conducted during a peak of the COVID-19 pandemic, we did not notice relevant correlations between GDF-15 serum levels and a positive COVID-19 status. The limited number of infected patients and the rather mild forms of the disease have possibly contributed to this finding, in contrast with the vastly incriminated role of severe acute respiratory syndrome coronavirus type 2 (SARS-CoV-2) infection not only in the intrinsic pathophysiology of cardiovascular diseases [48,49], but also in increasing the serum levels of cardiac biomarkers [50].

GDF-15 exhibited a solid diagnostic performance, similar to that of NT-proBNP, with the ROC-derived cut-off value of 313.9 ng/L being 75% sensitive and 77.6% specific in diagnosing acute HF. The interdependence between the two biomarkers was confirmed by a strong positive correlation (R = 0.264, *p* = 0.004), followed by a significant linear regression that mirrored the precise mutual variation. Consequently, we hypothesized that GDF-15 would be a valuable addition to the routinely determined NT-proBNP, not only for diagnostic purposes but also for improvement of risk stratification, even from admission.

The prognostic value of GDF-15 is underlined in multiple studies, which demonstrated the association of its high concentrations with increased mortality, not only in patients with HF or other cardiovascular pathologies [51,52], but also in other non-cardiac patients, or even in healthy controls, after 10 years of follow-up [53]. The role of GDF-15 in risk stratification also resides in its serial measurements, as an extensive study found that a 40% increase in GDF-15 levels was associated with a 4-fold increased risk of mortality [53]. In antithesis with this finding, Lourenço et al. reported that it is not the variation of GDF-15 (at admission versus discharge) that provides prognostic impact, but rather the intrinsic elevated concentrations would predict an increased risk of mortality [54]. In our research, we found a 6-fold higher mortality in patients with a GDF-15 concentration above 618 ng/L, which is considered a high-risk cut-off. However, this value is significantly lower than the 1800 ng/L cut-off, previously reported by Bonaca et al. as being associated with a poor prognosis [55]. However, these figures should be integrated into the clinical context of the patient, taking into account the ubiquity of GDF-15’s expression and the fact that it is upregulated in a variety of potentially fatal malignancies (e.g., pancreatic, gastric, ovarian, breast, melanoma, glioblastoma, etc.) or autoimmune diseases (e.g., rheumatoid arthritis). Nevertheless, the strict exclusion criteria of our study minimized the confounding influence of severe pathologies on GDF-15. In this context, a rational approach in real-life practice would be to correlate GDF-15 serum levels with both systemic and cardiovascular symptoms. Any discordance between a biomarker’s elevated concentration and a rather compensated clinical and echocardiographic presentation should raise the suspicion of another possibly severe underlying pathology, further guiding clinicians toward supplementary investigations for an integrative evaluation.

Given the fact that NPs are still considered the gold standard, the concept of a dual diagnostic-prognostic approach for patients with acute HF currently envisages the association between a classic biomarker (e.g., NT-proBNP) and a modern molecule, such as GDF-15, which has a superior prognostic value. Using Rehman’s study on ST2 as a model [56], by reporting the mortality rates according to the median concentrations of biomarkers, we noted that the combined use of GDF-15 with NT-proBNP acts synergistically and can predict mortality at 1 month significantly more accurately than individual determinations of each biomarker. Thus, the lowest mortality (7.3%) was recorded among patients with both biomarkers below the median values, followed by a 10.5% mortality rate when only NT-proBNP was elevated, and 25% when GDF-15 alone was increased. Basically, even isolated increases of GDF-15 were an independent and superior predictor of mortality, compared to patients with only isolated increases of NT-proBNP (25% versus 10.5% mortality rate, *p* < 0.05). Moreover, an even higher fatality rate (40%) was recorded when both GDF-15 and NT-proBNP were elevated, a finding consistent with previous results showing significant increases in mortality estimates based on composite models [56,57,58,59].

Ischemia due to acute coronary syndrome (ACS) is a common cause of acute HF or, at least, is a trigger that leads to the rapid decompensation of previously stable HF. In this regard, current guidelines recommend troponin assessment for excluding type I acute myocardial infarction (AMI) as a cause of acute HF, for this purpose being useful to corroborate biomarker results with clinical, ECG, or echocardiographic data. Although it is mainly a biomarker of myocardial necrosis and is useful in the diagnosis of AMI, elevated troponin serum levels are also found in both acute and chronic HF. In the acute setting (either de novo or chronic with acute decompensation), a high troponin has consistently been shown to correlate with increased mortality and readmission rates, thus contributing to the prognosis evaluation [60]. Multiple pathophysiological mechanisms may influence troponin elevation in acute HF, as well as in other underlying pathologies other than acute ischemia, including cardiomyocyte necrosis, apoptosis, cellular autophagy, membrane proteolysis, or increased membrane permeability [61].

In clinical practice, serial measurements of cardiac troponins are extremely useful: maintaining serum levels within normal limits 3–6 h after the clinical onset of symptoms suggestive of acute HF practically excludes AMI as its etiology and requires the continuation of the differential diagnosis algorithm. In our study, hs-troponin exhibited adequate sensitivity and specificity in differentiating between acute and chronic HF but without presenting a significant predictive value for a poor outcome.

In the context of these data, we consider that the rational approach for risk stratification in patients with acute HF is represented by the use of a multimarker panel—comprising both classic and modern biomarkers—that can provide complementary prognostic or pathophysiological information. However, there is still a lack of consensus regarding the “ideal combination” of biomarkers with a balanced cost/prognostic value ratio. In this sense, by including biomarkers directly correlated with a negative prognosis, we performed several multivariable regressions and observed that the inclusion of GDF-15 in a model that includes either only troponin (R = 0.438, R^2^ = 0.191), or both troponin and NT-proBNP (R = 0.451, R^2^= 0.203) represented the most appropriate predictive models in mortality risk assessment in patients with acute HF. Even if the intrinsic addition of NT-proBNP in the multimarker model is rather modest, it does not automatically imply the exclusion of it from risk stratification. It must be taken into account the great variability of NPs, dependent on certain coexisting pathological conditions. It has already been confirmed that obesity induces lower levels of NT-proBNP; this aspect, in conjunction with the significant percentage of 35% obese patients in our study, provides a reasonable explanation for a lower prognostic value compared to GDF-15, which is not influenced by such confounding factors [24,62].

As future perspectives, we can assume that a dynamic assessment of GDF-15, starting from admission, continued at discharge, and at the subsequent follow-ups, could not only improve the risk stratification but could also guide the treatment toward more potent medications and/or enhanced doses. Although GDF-15’s exact mechanism of action in HF has not yet been elucidated, it is a polymorphic biomarker that reflects a constellation of pathophysiological pathways, and its inclusion in a multimarker panel certainly adds incremental information concerning the initial approach of patients with acute HF.

### Limitations of the Study

The main limitations of the study were its unicentric design and the relatively limited number of enrolled patients. This situation mainly occurred in the context of the COVID-19 pandemic, especially since the study was mainly conducted during the third wave of the pandemic. Our hospital was designated as a support facility with rather restrictive conditions for enrollment procedures or follow-up visits. Additional restrictions referred to the assessment of biomarkers only at admission, with each patient assigned a single GDF-15 kit.

## 5. Conclusions

To our knowledge, this is the first report regarding the prognostic value of GDF-15 in patients with acute HF from Romania, if not from entire Eastern Europe. Therefore, we have endeavored to provide a detailed insight concerning the diagnostic and prognostic particularities of GDF-15 in patients presenting in emergencies with acute HF. In this regard, we noticed in the first place that the serum levels of GDF-15 were significantly higher among patients with acute HF compared to their stable, chronic counterparts. Moreover, GDF-15 also demonstrated consistent diagnostic performance, expressed as an AUC of 0.883, similar to that of the *gold standard* NT-proBNP. Concerning the prognostic value, GDF-15 was significantly correlated with validated markers of poor prognosis, being a superior mortality predictor compared to classical biomarkers (NT-proBNP and hs-troponin), both individually, or as part of a multimarker model.

We emphasize that high levels of GDF-15 were not significantly associated with an impaired LV ejection fraction or with an increased LV diameter; these aspects suggest the intricate pathophysiological pathways expressed by this biomarker, and not just the pressure or volume overload. Equally important, GDF-15 concentrations were not influenced by age, gender, or obesity, which are common confounding factors for other biomarkers, especially NT-proBNP.

These qualities exhibited by GDF-15 can change the classic paradigm concerning both the initial approach and the long-term prognostic assessment of patients with acute HF—a composite panel that includes novel and classical biomarkers emerging as a promising and feasible scenario. However, additional multicenter, extensive studies are further required to confirm GDF-15’s overexpression in patients with acute HF, possibly even identifying its precise underlying mechanisms.

## Figures and Tables

**Table 1:** Demographic and clinical characteristics.

Characteristics	Total (N = 173)			Acute HF (N = 120)			Control Group (N = 53)			*p*-Value
	Min	Mean ± STD	Max	Min	Mean ± STD	Max	Min	Mean ± STD	Max	
Age (years)	18	65 ± 13.3	94	18	66.4 ± 15.3	94	30	64 ± 11.9	85	0.526
Mortality rate: N (%)		21 (12.1%)			21 (17.5%)			0 (0%)		<0.001
Gender										0.438
Male: N, (%)		104 (60%)			71 (59.20%)			33 (62.30%)		
Female: N, (%)		69 (40%)			49 (40.80%)			20 (37.70%)		
Smoking: N, (%)		67 (38.7%)			48 (40%)			19 (35.8%)		0.605
Alcohol abuse: N, (%)		97 (56.1%)			75 (62.5%)			22 (41.5%)		0.012
Arterial hypertension: N, (%)		94 (54.3%)			60 (50%)			34 (64.2%)		0.085
Ischemic heart disease: N, (%)		76 (43.9%)			59 (49.2%)			17 (32%)		0.037
Diabetes mellitus N, (%)		29 (16.8%)			22 (18.3%)			7 (13.2%)		0.406
Obesity (BMI > 30 kg/m^2^): N, (%)		49 (28.3%)			42 (35%)			7 (13.2%)		0.003
Anemia N, (%)		47 (27.2%)			35 (29.2%)			12 (22.7%)		0.377
COVID-19: N, (%)		13 (7.5%)			8 (6.7%)			5 (9.4%)		0.241

Legend: N—number; BMI—body mass index; COVID-19-coronavirus disease 2019; STD—standard deviation.

**Table 2:** Echocardiographic parameters, laboratory data, and therapeutic aspectsw.

	Total (N = 173)			Acute HF (N = 120)			Control Group (N = 53)			*p*-Value
Characteristics	Min	Mean ± STD	Max	Min	Mean ± STD	Max	Min	Mean ± STD	Max	
LV ejection fraction (%)	10%	39.4 ± 14.4	72%	10%	33.8 ± 13.9%	61 %	38%	52.2 ± 15.7	72%	0.017
LV end-diastolic diameter (mm) N, (%)	30	52.7 ± 8.2	75	32	55.1 ± 8.6	75	30	47.3 ± 7.7	63	0.033
Hemoglobin (g/dL)	7.20	13.3 ± 2.1	33.7	7.20	13.2 ± 2.4	33.7	9.60	13.5 ± 1.9	18.40	0.069
Hematocrit (%)	22.20	39.5 ± 9.7	56.3	22.20	39.5 ± 8.9	56.3	29.2	39.4 ± 11.2	52.70	0.909
Leukocytes (×10^9^/L)	1.20	9.5 ± 1.3	30.2	4.10	10.2 ± 1.34	25.2	1.2	7.9 ± 1.2	30.18	0.044
Platelets (×10^3^/μL)	37	266 ± 43	2630	37	270 ± 45	2630	37	253 ± 42	595	0.245
Blood glucose (mg/dL)	63	142.5 ± 37.1	582	63	149.2 ± 33.4	582	73	128.1 ± 41.8	388	0.111
Total bilirubin (mg/dL)	0.09	1 ± 0.2	5.02	0.10	1.2 ± 0.2	5.02	0.1	0.5 ± 0.2	1.12	<0.001
Sodium (mmol/L)	121	138.3 ± 12.6	147	121	137.8 ± 14.1	147	122	141.1 ± 8.5	147	0.002
Potassium (mmol/L)	2.90	4.5 ± 0.8	6.3	2.90	4.6 ± 0.9	6.30	3.20	4.3 ± 0.7	5.60	0.009
Creatinine (mg/dL)	0.60	1.2 ± 0.3	4.0	0.60	1.2 ± 0.3	4.01	0.7	1 ± 0.2	3.78	0.029
Total cholesterol (mg/dL)	63	164.2 ± 51.2	331	63	161.1 ± 51.9	331	90	173.3 ± 49.3	285	0.109
LDL-cholesterol (mg/dL)	33	109.2 ± 33.4	255	33	106.6 ± 35.6	255	53	124.6 ± 31.2	197	0.253
HDL-cholesterol (mg/dL)	12	41.7 ± 16.5	111	12	40 ± 15.9	111	36	52.4 ± 17.7	76	<0.001
Beta-blockers		148 (85.6%)			99 (82.5%)			49 (92.5%)		0.087
Inhibitors of the RAS		121 (70%)			77 (64.2%)			44 (83.1%)		0.012
Loop-diuretics		119 (68.8%)			102 (85%)			17 (32.1%)		<0.001
Mineralocorticoid receptor antagonist		92 (53.2%)			83 (69.2%)			9 (17%)		<0.001

Legend: N—number; LV—left ventricle; LDL—low density lipoprotein; HDL—high density lipoprotein; RAS—renin-angiotensin system; STD—standard deviation.

**Table 3:** GDF-15 and the standard biomarker panel in patients with acute HF versus the control group.

Biomarker	Total = 173	Acute HF = 120	Controls = 53	*p*-Value
GDF-15 (ng/L)	439 (199–786)	596 (305–904)	216 (139–305)	<0.01
NT-proBNP (pg/mL)	3757 (1827–9764)	5440 (2812–12791)	107.80 (41.30–325.25)	<0.01
hs-Troponin (ng/L)	31.01 (7.03–104.80)	38.25 (12.45–179.50)	2.26 (1.14–5.43)	<0.01

Legend: GDF-15—growth differentiating factor-15; NT-proBNP—amino-terminal pro-B-type natriuretic peptide. Hs-Troponin—high-sensitive troponin. The results are expressed as medians (IQR 25–75).

**Table 4:** Correlations between GDF-15 serum levels and relevant parameters.

Parameter	GDF−15	
	r	*p*−Value
LVEF	−0.117	0.125
LVEDD	−0.021	0.873
LAVI	0.142	0.085
E/e’	0.191	0.038
sPAP	0.188	0.046
NT−proBNP	0.264	0.004
hs−Troponin	0.035	0.752
C−reactive protein	0.177	0.047
Hemoglobin	−0.095	0.101
Leukocytes	0.065	0.468
Serum creatinine	0.224	0.014
Sodium	−0.250	0.006
Potassium	0.165	0.03
Total bilirubin	0.376	<0.001
ALT	0.188	0.035
AST	0.302	<0.001
Total cholesterol	−0.423	<0.001
LDL−cholesterol	−0.370	<0.001
HDL−cholesterol	−0.242	0.011
BMI	−0.051	0.579
Age	0.166	0.07
Male gender	0.089	0.332
Alcohol abuse	−0.018	0.844
Smoking	0.070	0.499
Diabetes mellitus	0.170	0.063
Arterial hypertension	−0.056	0.545
Ischemic heart disease	0.016	0.861
Systolic blood pressure	−0.194	0.035
Diastolic blood pressure	−0.157	0.061
Heart rate	−0.031	0.755
Pulmonary crackles	0.306	<0.001
Peripheral edema	0.316	<0.001
Lactate level	0.301	<0.001
Inotropic support	0.256	0.005
Length of hospital stay	0.189	0.042
In−hospital mortality	0.283	<0.001
30−days mortality	0.375	<0.001

Legend: GDF-15—growth differentiation factor-15; LVEF—left ventricle ejection fraction; LVEDD—left ventricle end-diastolic diameter; LAVI—left atrial indexed volume; E/e’—transmitral early diastolic filling velocity/early diastolic LV myocardial velocity; sPAP—systolic pulmonary artery pressure; ALT—alanine transaminase; AST—aspartate transaminase; LDL—low density lipoprotein; HDL—high density lipoprotein cholesterol; BMI—body mass index.

**Table 5:** Detailed analysis of the area under curve (AUC): diagnostic performance of GDF-15 compared to NT-proBNP and hs-Troponin.

BIOMARKER	AUC	Std. Error	Asymptotic 95% Confidence Interval		*p*-Value
			Lower Bound	Upper Bound	
GDF-15 (ng/L)	0.883	0.028	0.828	0.938	<0.01
NT-proBNP (pg/mL)	0.976	0.013	0.952	1.000	<0.01
hs-Troponin (ng/L)	0.839	0.054	0.733	0.944	<0.01

**Table 6:** Relevant cut-off values for GDF-15.

Criterion	Concentration (ng/L)	Se	Sp
Se = Sp	306	0.750	0.755
Youden’s index (max Se + Sp)	314	0.750	0.776
High-mortality risk cut-off	618	0.483	0.867

Legend: Se—sensitivity; Sp—specificity.

**Table 7:** Overall survival assessment between subgroups according to the high-risk cut-off (618 ng/L).

	Chi-Square	df	*p*-Value
Log Rank (Mantel-Cox)	7.075	1	0.008
Test of equality of survival distributions for the different levels of GDF-15.			

**Table 8:** Synergistic effect of GDF-15 and NT-proBNP in predicting mortality rates.

Biomarkers		GDF-15 < 596 ng/L	GDF-15 > 596 ng/L	*p*-Value
	Mortality (%)			
NT-proBNP < 5440 pg/mL		7.3%	25%	<0.01
NT-proBNP > 5440 pg/mL		10.5%	40%	<0.01

**Table 9:** Multimarker model for predicting mortality rates in patients with acute HF.

Model	R	R^2^	Adjusted R^2^	Std. Error of the Estimate	*p*-Value	Durbin-Watson
	0.375 ^a^	0.141	0.133	0.385	<0.001	
1	0.438 ^b^	0.192	0.178	0.375	<0.001	
2	0.451 ^c^	0.203	0.183	0.374	<0.001	1.822

^a^. Predictors: (Constant): GDF-15 (*p* < 0.001). ^b^. Predictors: (Constant): GDF-15 (*p* < 0.001), hs-troponin (*p* = 0.007). ^c^. Predictors: GDF-15 (*p* < 0.001), hs-troponin (*p* = 0.018), NT-proBNP (*p* = 0.218). Dependent Variable: Mortality.

## Data Availability

Not applicable.
